# Global biogeography and ecological implications of cobamide-producing prokaryotes

**DOI:** 10.1093/ismejo/wrae009

**Published:** 2024-01-20

**Authors:** Jichen Wang, Yong-Guan Zhu, James M Tiedje, Yuan Ge

**Affiliations:** State Key Laboratory of Urban and Regional Ecology, Research Center for Eco-Environmental Sciences, Chinese Academy of Sciences, Beijing 100085, China; University of Chinese Academy of Sciences, Beijing 100049, China; State Key Laboratory of Urban and Regional Ecology, Research Center for Eco-Environmental Sciences, Chinese Academy of Sciences, Beijing 100085, China; University of Chinese Academy of Sciences, Beijing 100049, China; Center for Microbial Ecology, Michigan State University, East Lansing, MI 48824, United States; State Key Laboratory of Urban and Regional Ecology, Research Center for Eco-Environmental Sciences, Chinese Academy of Sciences, Beijing 100085, China; University of Chinese Academy of Sciences, Beijing 100049, China

**Keywords:** cobamide-producing prokaryotes, biogeography, ecological implications, metagenomic

## Abstract

Cobamides, a class of essential coenzymes synthesized only by a subset of prokaryotes, are model nutrients in microbial interaction studies and play significant roles in global ecosystems. Yet, their spatial patterns and functional roles remain poorly understood. Herein, we present an in-depth examination of cobamide-producing microorganisms, drawn from a comprehensive analysis of 2862 marine and 2979 soil metagenomic samples. A total of 1934 nonredundant metagenome-assembled genomes (MAGs) potentially capable of producing cobamides de novo were identified. The cobamide-producing MAGs are taxonomically diverse but habitat specific. They constituted only a fraction of all the recovered MAGs, with the majority of MAGs being potential cobamide users. By mapping the distribution of cobamide producers in marine and soil environments, distinct latitudinal gradients were observed: the marine environment showed peak abundance at the equator, whereas soil environments peaked at mid-latitudes. Importantly, significant and positive links between the abundance of cobamide producers and the diversity and functions of microbial communities were observed, as well as their promotional roles in essential biogeochemical cycles. These associations were more pronounced in marine samples than in soil samples, which suggests a heightened propensity for microorganisms to engage in cobamide sharing in fluid environments relative to the more spatially restricted soil environment. These findings shed light on the global patterns and potential ecological roles of cobamide-producing microorganisms in marine and soil ecosystems, enhancing our understanding of large-scale microbial interactions.

## Introduction

Cobamides are compounds with a cobalt corrin ring but differ in the identity of the lower ligand, and include vitamin B_12_ that plays a vital role in key metabolic processes in the majority of living organisms [1]. The de novo biosynthesis of cobamide involves more than 30 enzymatic steps (Supplementary Fig. 1), which can be separated into aerobic and anaerobic pathways, differing in their oxygen requirements and timing of cobalt insertion [2]. This is a high-genetic-burden, and probably as a result, it is exclusive to a restricted set of organisms capable of handling such complexity [3]. However, most microorganisms and all higher animals rely on cobamides [4, 5]. This suggests that the sharing of cobamides may be widespread across biology, which highlights the critical role of cobamide producers in ecosystems [6]. By providing cobamides, these producer organisms engage in interactions with cobamide-dependent ones, substantially influencing their growth. This results in the establishment of mutualistic symbiotic relationships [7], leading to the formation of intricate microbial ecological networks within ecosystems [8]. Accordingly, the exploration of cobamide-producing microbial members and their ecological roles in ecosystems bears considerable significance.

Cobamides are central to vital metabolic processes of life, including amino acid synthesis, DNA replication and repair, as well as energy metabolism [9]. The importance of cobamide-producing microorganisms in marine environments [10], soils [11], and hosts [12] is well-documented. A typical case is the metabolic dependency of algae on cobamides, with approximately half of the algae strictly requiring exogenous cobamide supply during growth. By symbiotically associating with cobamide producers, algae achieve cobamides and provide the carbon sources for the latter [13]. This enables the algae to carry out a series of cobamide-dependent enzymatic reactions, including the synthesis of methionine, ribonucleotide reduction, and the isomerization of methylmalonyl coenzyme A [14]. In mammals, including humans, aside from dietary supplementation, cobamide producers in the intestinal microbiota also supply cobamides to the host and the surrounding community. For example, in the human body, the synthesis of methionine, which depends on cobalamin, is closely connected to folate cycling, and a deficiency in cobalamin can lead to severe pathologies [15]. In addition to the provision of cobamides, these microbes also play critical roles in various biogeochemical processes [16]. The functions of cobamides in the cycling of carbon (C), nitrogen (N), and sulfur (S) have been documented, acting as cofactors in enzymatic processes or enhancing the growth of microorganisms [9, 17–19]. Moreover, some cobamide-producing microorganisms are themselves significant contributors to biogeochemical functions. Examples include the *Roseobacter* clades with extensive metabolic capabilities in N, phosphorus (P), and S cycling [20], the marine *Rhizobiales* known for their sulfate metabolism [21]; and the Thaumarchaeota, recognized for their ammonia oxidation abilities [7]. Furthermore, metabolic processes dependent on cobamides have catalytic and energetic advantages, which can influence the compositions of microbial communities and subsequently affect their ecological functions [22]. Nevertheless, the specific roles cobamide-producing microbes play in ecosystem functionality and the driving factors behind their ecological distribution remain underreported. Furthermore, we lack comparative insights into the ecological roles of cobamide producers in oceans and soils, Earth’s largest habitats. The former, a fluid ecosystem, primarily features algae in the photic zone—organisms mostly reliant on cobamides—for primary production. Whereas in soil, which has a more stable matrix, large plants serve as the primary producers and are independent of cobamides [13, 14]. The compositions of cobamide biosynthesis genes in global marine [23] and soil [11] environments have been previously reported; however, these studies primarily relied on gene analyses, leading to an incomplete understanding of the individuals and a deficiency of estimating biogeographic abundance in these two distinct habitats. Consequently, our study delves into the abundance of cobamide producers in both marine and soil environments. Considering the disparities of mobility of both microbial strains and substrates, we propose a hypothesis that the ratio of cobamide producers to users in marine environment would be lower than that in soil environment. Furthermore, taking into account the distinct requirements of cobamides for primary producers in marine and soil ecosystems, we hypothesize that cobamide producers in marine would exhibit a more pronounced association with microbial community and functions compared to those in soil environment.

The global application of shotgun metagenomic sequencing has become a pivotal tool for examining complex microbial communities [24], uncovering different taxa and functional capacities. Hence, we used the available datasets of metagenomes from global marine and soil ecosystems to produce metagenomic-assembled genomes (MAGs) and yielding the currently largest genomic database of prokaryotic cobamide producers, covering marine and soil. By comparing raw sequencing reads from metagenomic data with these genomes, we identified and quantified the abundance of cobamide producers in marine and soil environments, along with key habitat attributes that may influence their distribution. We mapped global predictions of cobamide producers’ abundance, highlighting hot spot regions within these environments. Lastly, we explored the roles of cobamide producers in these ecosystems by correlating their abundance with microbial community diversity, function, and biogeochemical cycling genes. Our findings enhance the understanding of cobamide-producing microbial ecology and the inference of their functionality within ecosystems.

## Materials and methods

### Collection of metagenomic samples

**Figure 1:**
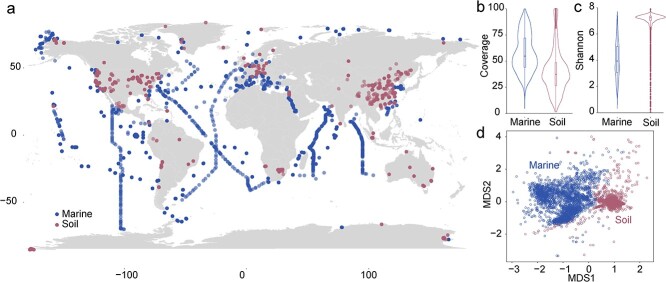
Geographical distribution of 2862 metagenomic samples from marine and 2979 samples from soil collected in this study (A); coverage of marine and soil metagenomic samples (B); Shannon index of marine and soil metagenomic samples (C); NMDS analysis of marine and soil metagenomic samples (D).

We collected 2979 soil metagenomic samples in June 2022, and 2862 marine metagenomic samples in August 2022 from the Sequence Read Archive (SRA) database (Fig. 1). In brief, the keywords of “soil” AND “metagenomics” were used for the soil dataset, and “marine” OR “ocean” AND “metagenomics” for the marine set to perform the primary search. For the reason that Illumina platform accounted for the majority in SRA database, therefore, only the metagenomes sequenced by the shotgun sequencing using the Illumina platform were chosen to avoid platform bias. To ensure the quality of data, sequences were required to have a higher average spot length value than 150, and a greater size than 500 M. As this study investigates the ecological behavior of microorganisms in natural environments, samples without geographic coordinates or collected from laboratory experiments were excluded. The SRA Toolkit (https://trace.ncbi.nlm.nih.gov/Traces/sra/sra.cgi?view=toolkit_doc) was used to download ~47 terabytes of SRA files and convert them to FASTQ format. The detailed information of these metagenomic samples is available in Supplementary Tables S1 and S2.

### Quality control and analysis of metagenomic samples.

Trimmomatic [25] was used to filter adapters, short reads, and low-quality reads of the downloaded raw paired sequences. The parameters were set as follows: LEADING:2, TRAILING:3, SLIDINGWINDOW:4:20, MINLEN:60, ILLUMINACLIP:2:30:10:8. Nonpareil 3 [26] was used to estimate the coverage of metagenomes. MEGAHIT v1.2.9 [27] was subsequently used to assemble contigs from the cleaned sequences of each sample using the default parameter. Prodigal v2.6.3 [28] was used to predict the open reading frames from the assembled contigs. Salmon v1.90 [29] with default parameters was employed to estimate the abundance of genes for each sample. To estimate the taxonomic classification and the relative abundance in each sample, Kraken2 v2.1.0 [30] and Bracken v2.6 [31] were used against the “Standard” database, only involving taxa classified to bacteria and archaea and removing viral manually. To calculate the Shannon and Richness indices, the total reads of assigned taxa for each sample were rarefied to the lowest sample using “rrarefy” function of vegan package of R language [32].

### Assembly of metagenome-assembled genomes

MaxBin v2.2.7 [33] was employed to assemble the contigs longer than 1000 bp for each metagenomic sample using default parameters. The CheckM v1.1.3 lineage_wf function [34], also with default parameters, was used to estimate the completeness and contamination of the recovered bins. A bin with a quality score (calculated as completeness – [5 × contamination]) [35] higher than 50 was considered a MAG and used for further analysis.

### Identification of cobamide potential producers and users

Processes for identifying potential producers of cobamide have been described in our previous study [36]. Briefly, Hidden Markov Model (HMM) profiles (Supplementary Fig. 1) for the genes involved in the key biosynthesis processes, according to Kyoto Encyclopedia of Genes and Genomes (KEGG) database [37], were obtained from the TIGRFAM [38] or PFAM [39] databases. Based on these profiles, the HMM search v3.3.2 [40] was used to detect the respective gene from the predicted proteins of each MAG, with the E-value cutoff set at 1 × 10^−6^, based on previous recommendations [11, 23]. For the shared orthologous genes between aerobic and anaerobic corrin ring pathways [5], the same HMM profile was used (Supplementary Fig. 1). We defined the MAGs into four different phenotypes according to the criteria shown in Supplementary Table S3: “very-likely-producer,” “likely-producer,” “possible-producer,” and “non-producer.” The MAGs defined as very-likely-producers, likely-producers, or possible-producers were considered as potential cobamide producers (Supplementary Tables S4 and S5). They were taxonomically classified using the Genome Taxonomy Database Toolkit (GTDB-Tk) v1.7.0 [41] (Supplementary Table S6) and were dereplicated at a 95% similarity threshold using dRep v3.2.2 [42]. GTDB-Tk was also used with the functional module “infer” to construct bacterial and archaeal trees for the dereplicated MAG producers, which were then visualized using IToL v6.5 [43]. METABOLIC-G v4.0 [44] was used to analyze the presence/absence of metabolic pathways in each MAG from marine and soil environments. According to a previous study [5], 13 metabolic processes that are dependent on cobamide were selected according to the respective HMM profile using HMM search v3.3.2 (Supplementary Table S7). A MAG was considered as a potential cobamide user if it contained one or more of these enzyme families.

### Quantification of cobamide biosynthesis genes and producers

The same HMM profiles and parameters were used in an HMM search against the proteins that predicted from assembled contigs to estimate the read counts of cobamide biosynthesis genes of each metagenomic sample. The relative abundance of each gene in each sample was calculated by normalizing the detected read numbers to the reads per kilobase of exon model per million mapped reads (RPKM) by combining the results of gene abundance from Salmon. SeqKit toolkit [45] was utilized to extract the matches, and these sequences were further annotated using Kraken2 v2.1.0. To estimate the abundance of cobamide producers in a shotgun metagenome, we initially constructed separate databases for soil and marine samples. We searched for 40 universal single-copy gene families commonly used to recruit metagenomic reads [46] against cobamide producer MAGs using *hmmsearch* [40]. Referring to the method of Nayfach’s study [47], we selected the top 15 coverage gene families (higher than 85.4% for marine and 85.6% for soil; Supplementary Table S8) detected from these MAG, and the gene sequence with the highest score for each MAG was used to construct the databases for marine or soil samples based on their respective producer MAGs. Subsequently, we employed Salmon to estimate the read counts of each sample that mapped to the database and normalized them to RPKM. According to the taxonomic classification of these MAGs, we calculated the relative abundances of different taxa of cobamide producers in each sample.

### Mapping global cobamide producer abundance

We constructed random forest models [48] separately for marine and soil cobamide producer abundance. We initially prepared worldwide rasters of environmental variables from public databases. A total of 18 marine variables and 15 terrestrial variables (Supplementary Table S9) associated with microorganisms were utilized to predict the global distribution of marine and soil cobamide producer abundance, respectively. For the reason that metagenomes were derived from different studies, to avoid our model from excessively projecting beyond its training dataset, potential biases (443 samples for soil, 62 samples for marine) were mitigated by excluding outliers only once using the *boxplot.stats* function of R for both marine and soil models. We also considered the impact of sample depth on cobamide producer abundance (Pearson’s *P* < .001, *n* = 1885, *r* = 0.42 for marine samples). We found that the influence of marine depths above 100 m was not significant (Pearson’s *P* = .559, *n* = 1580, *r* = 0.02), therefore 304 metagenomes collected from depth below 100 m were excluded from the random forest model of marine samples. Metagenomes lacking depth information or no clear indication of deep layer were considered surface samples and included in the analysis. Similarly, 57 metagenomes collected from soil depths exceeding 30 cm were removed from the soil model, whereas those lacking depth information or with no clear indication of deep layer were treated as surface samples and included in the analysis.

The hyperparameter tuning for random forest model was conducted based on grid search to determine the best combination of hyperparameters. The procedures for feature selection and parameter tuning were performed using k-fold cross-validation (k = 10 in the present study) to ensure that the test sets were independent of the training sets and to minimize the potential for model overfitting. The cross-validated root mean square error value was used to identify the best hyperparameters. Using these optimal parameters of ntree, mtry, and the number of variables (identified using recursive feature elimination algorithm) for both marine and soil models (Supplementary Figs 2–4), predictions were carried out at a global scale (0.2° × 0.2°). The uncertainty tied to these predicted values was also quantified and documented (Supplementary Fig. 5). Random forest analyses were performed using “randomForest” [49] and “caret” [50] packages of R language. Predicated values were visualized in global maps using “ggplot2” package of R language.

### Analysis of functional profiles and nutrient cycle processes

To estimate the abundance of metabolic pathway functions, we utilized HUMAnN 3.5 [51] on the clean raw fastq reads of each sample. Default parameters were set, and the UniRef90 and ChocoPhlAn databases were used. The relative abundance of pathways for each sample was normalized using the accompanying script *humann*_*renorm*_*table*. The relative abundance of nutrient cycle processes of N, S, and P was analyzed, utilizing the databases and tools of NCycDB [52], SCycDB [53], and PCycDB [54], respectively. According to the instructions of NCycDB and SCycD, raw clean reads were aligned to the available databases using diamond v2.1.6 [55], and the relative abundance of each gene was normalized to a designated number of sequences for random subsampling. For the PCycDB tool, all assembled reads from each sample were first aligned to the “nr” database using diamond v2.1.6, followed by a “filter” step to identify P-cycling genes using the attached script *filter_Generate_ORF2gene.py*. The relative abundance was further calculated by combining the results of Salmon using the attached script *Coverage_get.py*. The default parameters were set according to the recommendations of these three tools.

### Statistical analyses

All statistical analysis were performed based on R language (version 3.6.1 or 4.1.3). Specifically, to show the differences of microbial communities between marine and soil samples, nonmetric multidimensional scaling (NMDS) analysis was performed using the vegan package based on the relative abundance of different species, and Permutational multivariate analysis of variance (PERMANOVA) was used to indicate a statistical confidence. Volcano plots were created to demonstrate the differences of discovery rates between metabolic pathways of cobamide producer MAGs and nonproducer MAGs, based on Wilcoxon tests with false discovery rate (FDR)-adjusted *P*-values and the Log_2_-fold change value of each metabolic pathway between the two groups. Linear regression analyses were conducted to estimate the influence of cobamide producers (Log_2_-transformed) on the Shannon and Richness indices of the entire microbial communities. To estimate the associations of cobamide producers and the *β*-diversity of microbial communities, Mantel tests were performed based on the Euclidean distance of the abundance of cobamide producers (Log_2_-transformed) and Bray–Curtis or Euclidean distance matrix of the relative abundance of species, using the vegan package. The UpSetR package was used to count the number of MAGs for each metabolic process dependent on cobamide. Spearman’s correlation analyses were performed to test the relationships between the abundance of cobamide producers (Log_2_-transformed) and the relative abundance of functional profiles or nutrient cycle processes, and FDR was used to adjust the *ρ*-values. The relative abundance of the whole N, S, or P cycles, as well as their separate processes, was calculated by summing all the scaled genes’ relative abundance, respectively.

## Results

### Cobamide producers are taxonomically diverse and habitat specific

**Figure 2:**
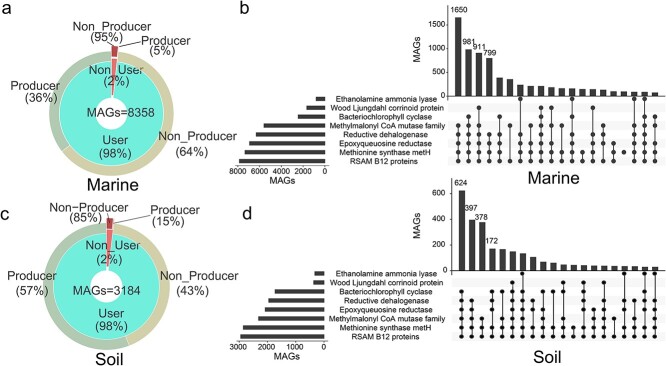
Proportion of potential producers and users of cobamides of metagenome-assembled genomes (MAGs) recovered from marine (a) and soil (c) environments; upset plots for number of MAGs that contain the pathways (eight most abundant) dependent on cobamides of marine (b) and soil (d) samples.

A total of 8358 and 3184 MAGs with genome quality score above 50 were recovered from 2862 marine and 2979 soil environments, respectively. These MAGs were grouped into four categories of cobamide producing bacteria: very-likely producer, likely-producer, possible-producer, or nonproducer. MAGs in the first three categories were considered potential producers of cobamides. A total of 2992 MAGs from marine and 1795 MAGs from soil were identified as potential producers of cobamides, which accounted for 35.8% and 56.4% of all the recovered marine and soil MAGs, respectively (Fig. 2a and c). The obtained results provided support for our first hypothesis, indicating that the proportion of cobamide producers in marine was indeed lower than that in soil environment. Among the marine samples, we identified 500 MAGs as Very-Likely producers, 1017 MAGs as Likely producers, and 1475 MAGs as Possible producers (Supplementary Table S4). In the soil samples, we identified 220 MAGs as Very-Likely producers, 515 MAGs as Likely producers, and 1060 as Possible producers (Supplementary Table S5). By identifying these cobamide-producing MAGs, we gain a more comprehensive understanding of their ecological distribution. More importantly, these MAGs serve as a valuable resource for conducting direct investigations into related microbial strains, thereby facilitating more targeted and informed studies.

**Figure 3:**
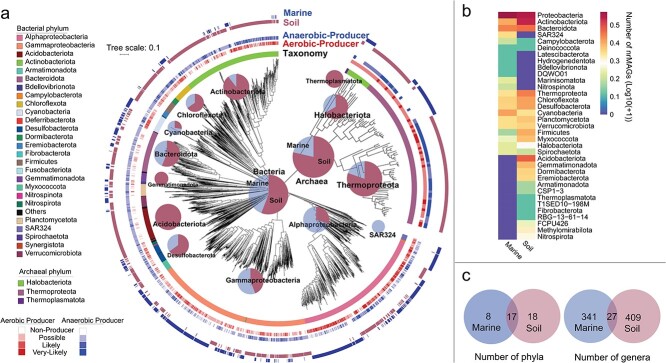
Phylogenetic trees of 1934 potential cobamide producer nrMAGs of bacteria (lower left) and archaea (top right) using GTDB-Tk); pie plots in phylogenetic trees show the proportion of these cobamide producer nrMAGs of marine and environments for bacterial and archaeal kingdoms and representative phyla; the information of identified four different phenotypes (“very-likely-producer,” “likely-producer,” “possible-producer,” and “non-producer”) for aerobic and anaerobic biosynthetic pathways and habitat for each MAG is shown in the phylogenetic trees (a); number of nrMAGs (Log10(+1) transformed) in marine and environments at phylum level (b); Venn plots show the number of phyla (left) or genera (right) in two environments (c).

All cobamide producer MAGs obtained from soil and marine environments were dereplicated as 1934 nonredundant MAGs (nrMAGs). These cobamide producer nrMAGs were high degree of taxonomic diversity with classifications covering 43 phyla and 777 different genera (Fig. 3a and Supplementary Table S10). These findings suggest that cobamide producers are not confined to specific taxonomic groups but are distributed across various lineages.

As might be expected, cobamide producers displayed habitat specificity. Only 17 phyla and 27 genera were found in both marine and soil environments (Fig. 3c). Certain phyla were exclusive to specific habitats, for instance, *Acidobacteriota* were found only in soil, while SAR324 were exclusive to marine environment (Fig. 3a). Furthermore, the predominant phyla of cobamide-producing nrMAGs varied across habitat. In marine environment, most nrMAGs were classified as *Proteobacteria* (68.9%) and *Bacteroidota* (6.9%), whereas in soil, *Proteobacteria* (30.0%) and *Actinobacteriota* (24.2%) were predominant.

### Global maps of cobamide producer abundance

**Figure 4:**
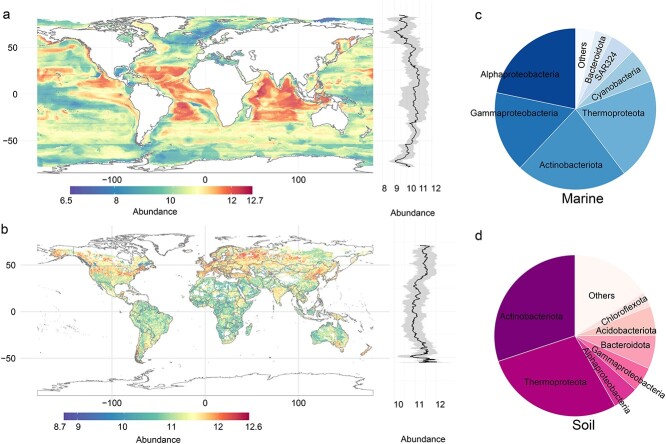
Global map of abundance of cobamide producers in marine (a) and soil (b) environments; the subfigures in right panels show the latitudinal variation of cobamide producer abundance across the global scale; the six most abundant phyla of cobamide producers in marine (c) and soil (d) environments; potential biases were mitigated by excluding outliers once only; samples in deep marine layers (> 100 m) and soil layers (> 30 cm) are not included; *Proteobacteria* is divided into its classes.

To quantify the relative abundance of cobamide producers in marine and soil habitats from a large number of metagenomes, we mapped metagenomic sequences with a database consisting of 15 universal single-copy genes from cobamide producers of marine or soil samples. In addition, the abundance of each gene listed in cobamide biosynthesis pathway (Supplementary Fig. 1) for each metagenomic sample was also estimated (Supplementary Fig. 6). Overall, the relative abundance of cobamide producers in surface soils (≤ 30 cm) was significantly higher than that of the marine surface (≤ 100 m) (Supplementary Fig. 7; Wilcoxon test, *P* < .001). Regarding the taxonomic compositions of surface marine samples (Fig. 4c), *Proteobacteria* (specifically, *α*- and *γ*- classes) were the predominant phylum of cobamide producers, comprising 40.0% of the total, followed by *Actinobacteriota* (22.3%) and *Thermoproteota* (20.3%). These results closely mirrored the patterns of abundance observed for cobamide biosynthesis genes (Supplementary Fig. 6). As for the surface soil samples (Fig. 4d), *Actinobacteriota* were the most prevalent phylum of cobamide producers, accounting for 30.1% of the total cobamide producers, followed by *Thermoproteota* (28.1%) and *Proteobacteria* (10.1%). These findings differed slightly from the pattern of cobamide biosynthesis gene abundances, where *Proteobacteria* contained more abundant biosynthesis genes than *Thermoproteota* (Supplementary Fig. 6). To test the possible bias due to the incompleteness or containment of MAGs, we employed cobamide-producing MAGs with a genomic completeness exceeding 90% and containment below 5% (comprising 1484 MAGs for marine environments and 971 MAGs for soil environments) to construct their respective databases and estimate the abundance of cobamide producers. The estimated abundances derived from these two databases exhibited a significant correlation (*P* < 2.2 × 10^−16^; Supplementary Fig. 8).

Random forest algorithm was used to generate predictive datasets to provide global spatial insight into the abundance of cobamide producers in the top layers of marine and soil environments (Fig. 4). Our marine mapping results revealed a noticeable latitudinal gradient (Supplementary Fig. 9) and regional variation in the abundance of cobamide producers (Fig. 4a), with the highest abundance observed in the equatorial regions of the Atlantic and Indian Oceans. In contrast, lower abundance was found in polar regions, with the lowest levels recorded in regions of the Antarctic Peninsula and Norwegian Sea. Our soil mapping analysis also demonstrated a latitudinal trend in the abundance of cobamide producers, with the lowest levels observed near the equator and highest levels at mid-latitudes that peaked at around ±40° latitude (Fig. 4b and Supplementary Fig. 10). On a regional scale, the abundance of cobamide producers was highest in areas of Europe, the northern regions of the USA, and northeastern China. Conversely, lower levels of cobamide producers were observed in locations like the Amazon Rainforest and Africa. Besides the geographical (latitudinal) influence on the abundance of cobamide producers, climatic factors like precipitation and temperature, environmental attributes such as pH, and nutrients also appeared to drive the variations of cobamide producers (Supplementary Figs 4, 9, and 10).

### Cobamide producers are potentially multifunctional beyond supplying cobamides

**Figure 5:**
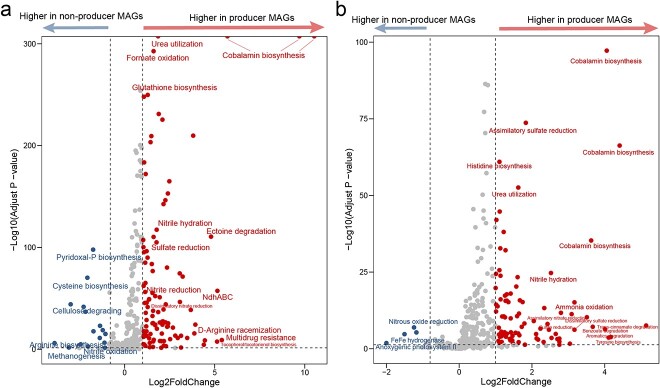
Volcano plots show difference between cobamide producer metagenome-assembled genomes (MAGs) and nonproducer MAGs (excluding nonusers) in marine a) and soil (b) environments, based on the presence or absence of a metabolic pathway in each MAG; the horizontal axis represents the fold change (Log_2_-transformed) of cobamide producer MAGs compared with nonproducer MAGs; the vertical axis represents the FDR-adjusted *P*-value; circles in right pane indicate a significant increase (fold change >1 and adjusted *P*-value <0.05) in the producer MAGs, whereas circles in the left pane indicate a significant decrease; labels of some typical pathways are highlighted; full results are available in Supplementary Tables S11 and S12.

To investigate the metabolic interdependencies between two cohorts of cobamide producers and nonproducers (excluding nonusers), metabolic pathways within each MAG were identified. Contrary to our expectations, cobamide producers also exhibited not only higher discovery rates of metabolic pathways related to cobamide synthesis, but also multiple other metabolic processes, such as urea utilization, amino acid biosynthesis, and others; this pattern was consistent across different habitats (Fig. 5 and Supplementary Tables S11 and S12). In addition, the genomic sizes of cobamide producer MAGs were significantly higher than that of nonproducer MAGs in both habitats (Wilcoxon test’s *P* < .01; Supplementary Fig. 11). Cobamide producers showed comparatively fewer missing metabolic pathways, including the biosynthesis of pyridoxal-P and cysteine in marine environments, nitrous oxide reduction, and FeFe hydrogenase in soil environments. Moreover, cobamide producers had increased nutrient cycling pathways for N and S, such as sulfate reduction and ammonia oxidation. Therefore, we further explored the potential ecological roles of cobamide producers in marine and soil ecosystems.

**Figure 6:**
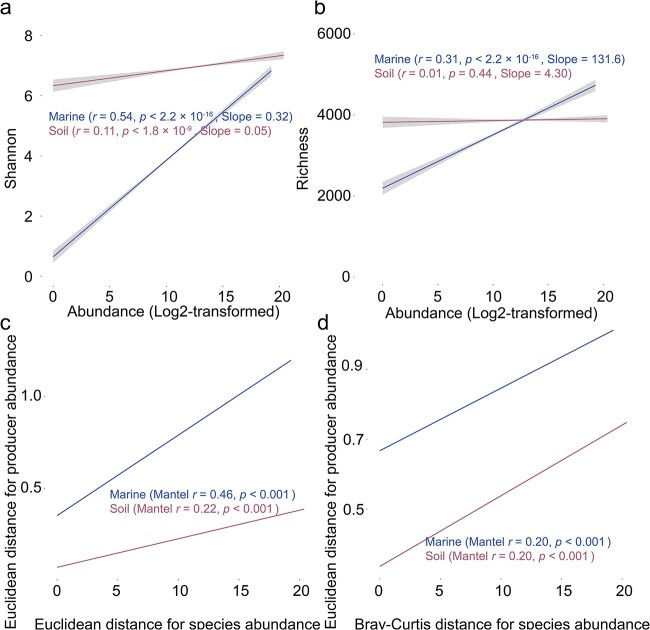
Regression analyses displaying the relationships between the abundance of cobamide producers (Log_2_-transformed) and Shannon (a) and richness (b) indices; Mantel tests show the relationships between Euclidean distance of cobamide producer abundance and beta diversity of community dissimilarity (calculated by the relative abundance of species) using Euclidean distance (c) and Bray–Curtis distance (d).

**Figure 7:**
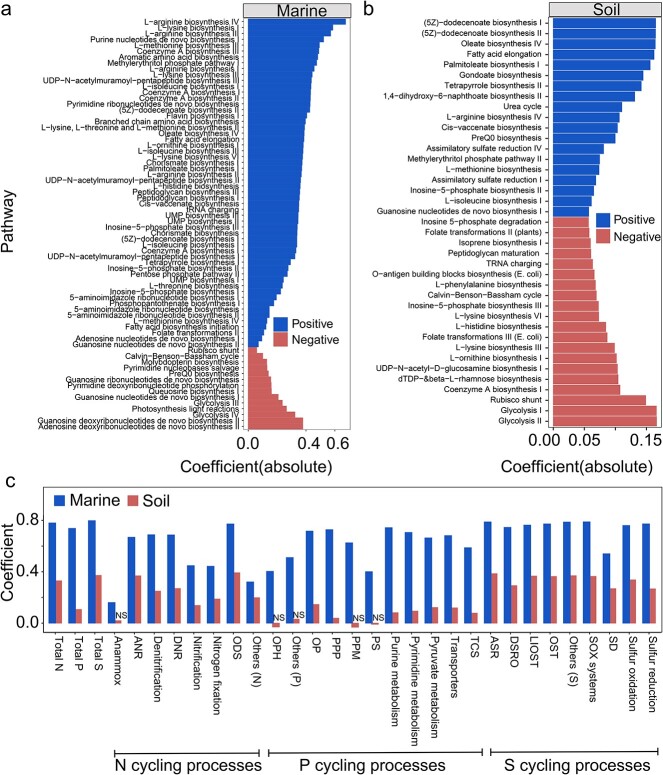
Spearman’s coefficients show the relationships between the abundance of cobamide producers (Log_2_-transformed) and the relative abundance of functional pathways in marine (a) and soil (b) environments; Spearman’s coefficients show the relationships between the abundance of cobamide producers (Log_2_-transformed) and the relative abundance of nitrogen (N), phosphorus (P), and sulfur (S) cycling processes (c); ANR assimilatory nitrate reduction, DNR dissimilatory nitrate reduction, ODS organic degradation and synthesis, OPH organic phosphoester hydrolysis, OP oxidative phosphorylation, PPP pentose phosphate pathway, PPM phosphonate and phosphinate metabolism, PS phosphotransferase system, TCS two-component system, ASR assimilatory sulfate reduction, DSRO dissimilatory sulfur reduction and oxidation, LIOSP linkages between inorganic and organic sulfur transformation, OST organic sulfur transformation, SD Sulfur disproportionation; NS represents not significant (*P* > .05).

Our results indicate a positive correlation between the abundance of cobamide producers and community indices of *α*- and *β*-diversity in both marine and soil habitats (Fig. 6) , and it is noteworthy that the associations were stronger in the marine ecosystem, as evidenced by higher values for slope and *R* [2]. Furthermore, we found a significant link between cobamide producers and certain community functions, particularly the biosynthesis pathways of some metabolites (Fig. 7a and b). In the marine environment, more positive relationships were observed than negative relationships, whereas an approximately even split was observed in the soil environment.

In the context of nutrient cycling processes involving N, S, and P, our findings revealed a significant and positive correlation between the abundance of cobamide producers and the majority of microbial N and S cycling processes in both marine and soil environments (Fig. 7c). In addition, although there was also a correlation with P cycling processes in the marine environment, this relationship was weaker in the soil environment.

## Discussion

Here, we describe the global distribution and potential functions of cobamide producers in marine and soil habitats. Consistent with a previous study conducted by Shelton *et al*. who surveyed over 11 000 bacterial species [5], our study found that cobamide-producing MAGs comprise a restricted set of the total community, whereas the majority of microorganisms host at least one enzyme family dependent on cobamides. Considering the higher catalytic and resource efficiency of cobamide-dependent metabolic processes compared with the independent ones [56, 57], it is reasonable to infer that cobamides are commonly shared among microorganisms [8]. Furthermore, in addition to providing cobamides, producers should also derive benefits from the recipients [58]. Our results showed that the cobamide producer MAGs have higher discovery rates of various metabolic pathways compared with that of nonproducer MAGs. We offer three plausible scenarios.

The first scenario is that the biosynthesis of cobamides is also dependent on other metabolic pathways, such as glutamate and *δ*-aminolevulinate [59], which could explain the high discovery rates of certain amino acid biosynthetic pathways. Second, it is possible that producers are restricted to obtaining carbon and energy sources from their recipients, such as the mutualistic relationship between cobamide producers and phototrophic microorganisms [13]. Alternatively, different species of cobamide-producing microorganisms may require various metabolites from their partners. These dependencies might not be observed through the analysis of metabolic processes and statistical tests utilized in the present study. Third, based on the analysis of a substantial number of metagenomic samples, we are confident in the robustness of our results that the cobamide producers have more functions beyond cobamides for microbial ecosystems. For example, the phylum *Thermoproteota* is known to be involved in ammonia oxidation [60]. Our present study, along with previous research [11, 23], reveals that they constitute a substantial proportion of total cobamide producers in marine and soil environments. In addition, the large genomic sizes of producer MAGs (Supplementary Fig. 11) can provide more genes [61], which supports the hypothesis mentioned above. This was further confirmed by the observed positive correlations between cobamide producers and community *α*- and *β*-diversity indices. Various metabolic pathways and processes related to N, P, and S cycling were also significantly correlated with cobamide producers, in addition to our findings revealed that cobamide producers are also potential functional microbes in multiple nutrient cycling processes. Cobamides are important cofactors in enzymatic processes of nutrient cycling or basic metabolic processes of these functional microbes [9, 17–19]. Moreover, prokaryotes that available of producing cobamides connect with the living things dependent on cobamides, they build a mutualistic relationship and form a complex connecting network [36], which potentially activates the diversity and improves metabolic functions and nutrient cycling of ecosystems [58]. These associations are more pronounced in marine samples compared to soil samples, suggesting that microorganisms in fluid environments are more inclined toward cobamide sharing than those in more static environments, supporting our second hypothesis. As a dynamic environment, substance exchange in marine ecosystems is facilitated, and the exchangeable spatial range is broader, both horizontally and vertically. In this study, the observation of fewer cobamide producers but more users in marine environments compared to soil environments provides strong evidence for the varying degrees of substance exchange by microorganisms in these two distinct types of environments. Importantly, over half of the primary producers in the ocean, such as algae, require a supply of cobamides, whereas plants, the primary producers in the soil ecosystem, do not directly need cobamides [62]. This significant difference in cobamide dependence implies that the importance of cobamides for marine ecosystems exceeds that of soil ecosystems.

This study produces quantitative maps of the surface of marine and soil habitats by assessing the abundance of cobamide producers. Furthermore, by integrating spatial covariates, the potential driving factors influencing these microorganisms were elucidated. Similar to the distribution pattern of bacterial diversity in previous studies of global ocean [63] and topsoil [64], the abundance of cobamide-producing prokaryotes also exhibits a clear latitudinal gradient. This indicates that cobamide producers are influenced by global-scale geographical differences, similar to the overall microbial community. Specifically, climate factors such as temperature [65], nutrient status [66, 67], and environmental conditions such as pH [68, 69] play significant roles in determining the distribution of these microbes. However, specific regions may show variations. For example, a previous study suggested that the areas with the highest marine diversity globally are regional peaks in Southeast Asia of the Pacific Ocean [70], whereas the abundance of cobamide producers peaked at the mid-Atlantic and Indian Ocean. This suggests that the microbial community of cobamide producers is driven by different factors at the regional scales, or possibly differentiated due to seasonal variations and ocean currents [71–73]. In addition to the shaping role of latitude, as revealed by random forest models, characteristics of marine and soil also had important roles in driving the patterns of cobamide producer abundance. Although the specific mechanisms may be complex, warm seawater temperature, high oxygen concentration, high photosynthesis due to available radiation, and high pH in marine environments might promote the enrichment of cobamide producers (Supplementary Fig. 9). The driving factors for cobamide producers in the soil environment are more complex (Supplementary Fig. 10). Generally, high annual solar radius, relative warm temperature, high contents of soil total N and organic P, and neutral soil pH tend to increase the abundance of soil cobamide producers. The results of these key driving factors provide support to explore the ecological niches of producing cobamides in marine and soil environments.

A precise evaluation of the global distribution patterns of cobamide producers was achieved through mapping metagenomic reads to the constructed databases. These results, although generally consistent with those obtained by directly comparing total gene abundance, exhibit some notable discrepancies. For example, the *β*-*Proteobacteria* are absent in the total abundance assessment but demonstrates a high cobamide synthesis gene abundance (Supplementary Fig. 6). This suggests that these microbes possibly do not possess a complete pathway for cobamide synthesis so are more likely to utilize salvage strategies [74–76]. Furthermore, it is important to consider potential biases due to differences between the two methods, namely potential overestimation of species abundance due to multiple cobamide synthesis genes in a single cell (average 2.74 HMM hits for each gene in a MAG). For instance, marine cyanobacteria demonstrate a high relative abundance of cobamide biosynthesis genes (37%), yet their species abundance constitutes only 10% of the total cobamide producers (Fig. 4c). Although comparing the community composition of cobamide producers by constructing a database using MAGs may offer a more accurate assessment than only analyzing the abundance and species composition of genes, it is crucial to alert researchers that the definition of cobamide producer MAGs and the quality of MAGs must be considered. In this study, the database included three categories of cobamide producers due to the concerning of the incomplete assembly of MAGs and the possibility of contamination. We also provided a higher quality MAG database to assess the abundance of cobamide producers, and the results from the two databases showed a significant positive correlation. However, there was still some bias when using two databases (Fig. S8). Therefore, researches can use different MAG criteria and definitions for cobamide producers based on their specific research conditions and use our shared MAGs to construct their databases. It is also worth mentioning that the predicted maps of cobamide producer distribution may not be accurate at fine spatial scales but can provide patterns at larger scales due to variations caused by the environmental heterogeneity of specific sites [48].

Our global mapping of cobamide producers’ abundance has revealed intriguing regional variations, potentially indicative of the influence of environmental factors on the distribution of these microorganisms. Moreover, our work highlights that through mutualistic relationships, cobamide producers might potentially improve the diversity and multifunctionality of microbial ecosystems.

## Conflicts of interest

The authors declare that they have no known competing financial interests or personal relationships that could have appeared to influence the work reported in this paper.

## Funding

This work was supported by the National Natural Science Foundation of China (42307162, 42177274), the Second Tibetan Plateau Scientific Expedition and Research Program (2019QZKK0308 and 2019QZKK0306), US National Science Foundation grant (DBI-1759892). This work was supported through computational resources and services provided by the Institute for Cyber-Enabled Research at Michigan State University.

## Data availability

All MAGs are available in Science Data Bank (https://doi.org/10.57760/sciencedb.08825; DOI: 10.57760/sciencedb.08825 for marine, and https://doi.org/10.57760/sciencedb.08826; DOI: 10.57760/sciencedb.08826 for soil). All secondary derived data and the scripts are available on GitHub (https://github.com/YuanGe-Lab/Jichen-Wang).

## Supplementary Material

Supp_Figures-R1_wrae009

Supplementary_Tables_wrae009
