# Supplementary material for: Global biogeography and ecological implications of cobamide-producing prokaryotes
Source: ISME J. 2024 Jan 20;18(1):wrae009. doi: 10.1093/ismejo/wrae009 (PMC10900890; doi:10.1093/ismejo/wrae009)
Supplement: Supp_Figures-R1_wrae009 [file supp_figures-r1_wrae009.pdf]

Supplementary Materials

Title: Global biogeography and ecological implications of cobamide-producing prokaryotes

Author Names: Jichen Wang[1,2], Yong-Guan Zhu[1,2], James M. Tiedje[3*], Yuan Ge[1,2*]

Author Affiliations:

1. State Key Laboratory of Urban and Regional Ecology, Research Center for Eco-Environmental Sciences, Chinese Academy of Sciences, Beijing 100085, China

2. University of Chinese Academy of Sciences, Beijing 100049, China

3. Center for Microbial Ecology, Michigan State University, East Lansing, MI 48824, United States

[*] **Corresponding authors:**

James M. Tiedje, Center for Microbial Ecology, Michigan State University, East Lansing, MI 48824, United States. Email: tiedjej@msu.edu.

Yuan Ge, Research Center for Eco-Environmental Sciences, Chinese Academy of Sciences, 18 Shuangqing Road, Beijing 100085, China. Tel: (86) 10 62913536, E-mail: yuange@rcees.ac.cn.

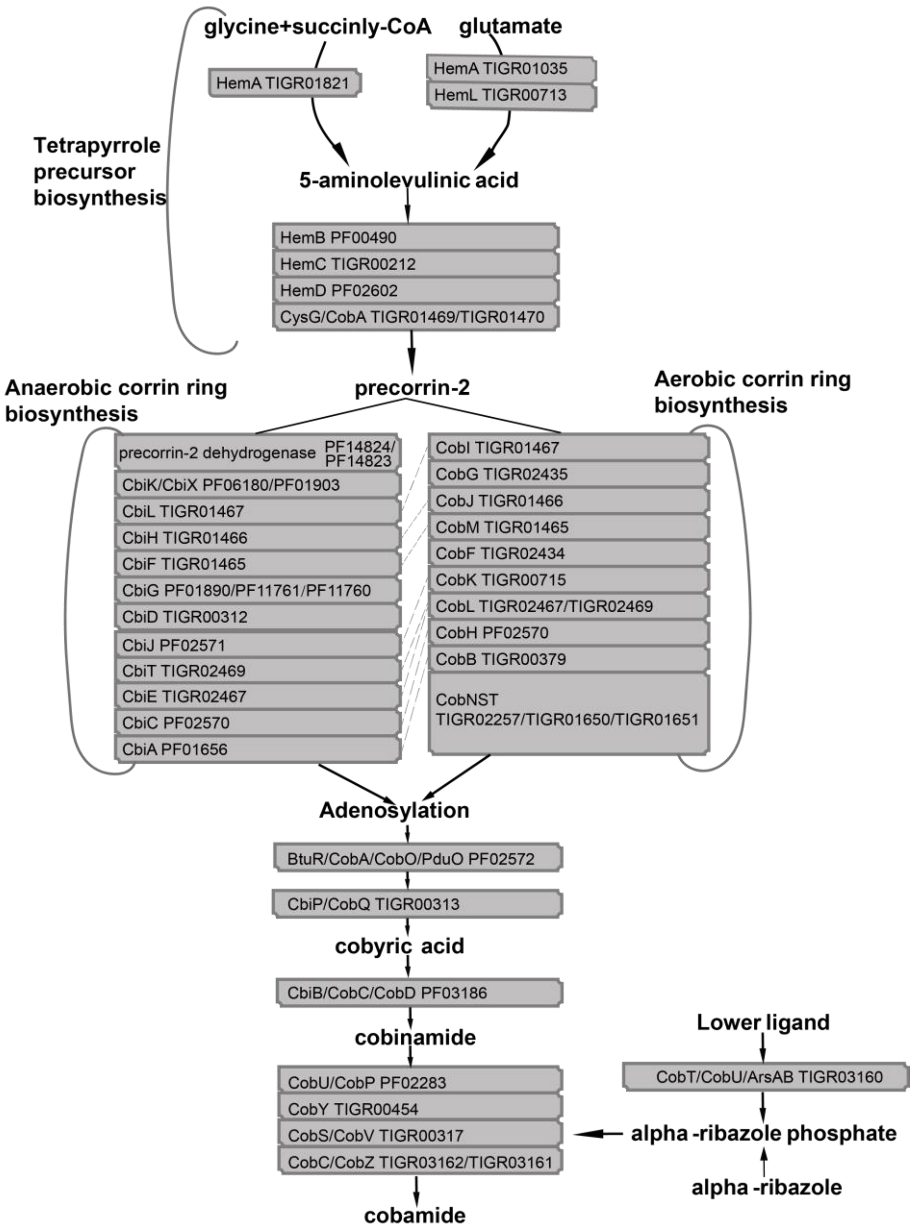

Supplementary Fig. 1 Cobamide biosynthesis with aerobic and anaerobic pathways. The cobamide biosynthesis pathway is shown with each enzymatic step indicated by a grey box labeled with the gene names and the profile HMMs (refer to TIGR or PFAM HMM numbers for detailed information). Dashed lines indicate orthologous enzymes for similar reactions in aerobic and anaerobic corrin ring biosynthesis.

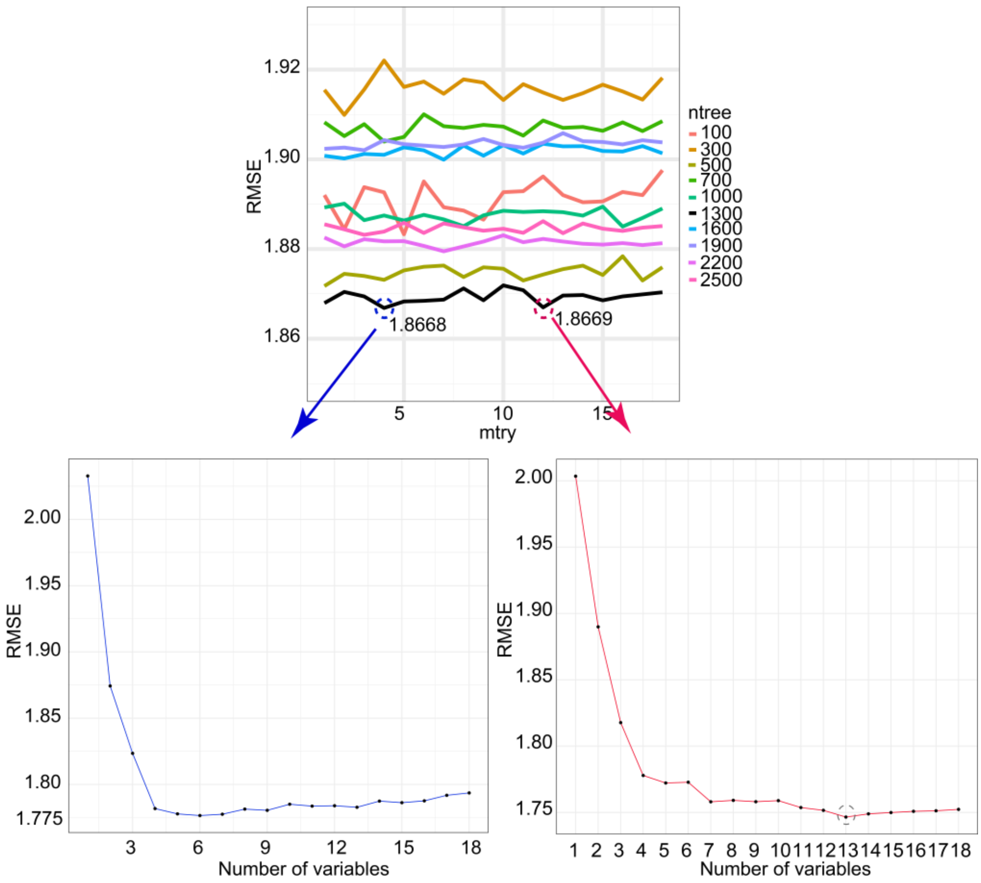

Supplementary Fig. 2 Hyperparameter tuning for random forest algorithm to predict the abundance of cobamide producers in marine environment based on grid search and 10-fold cross-validation. The best ntree is 1300, while mtry of 4 and 12 have very close lowest cross-validated root mean square error (RMSE) values. The following cross-validations show that the best parameters of ntree, mtry, and number of variables are 1300, 12, and 13, respectively.

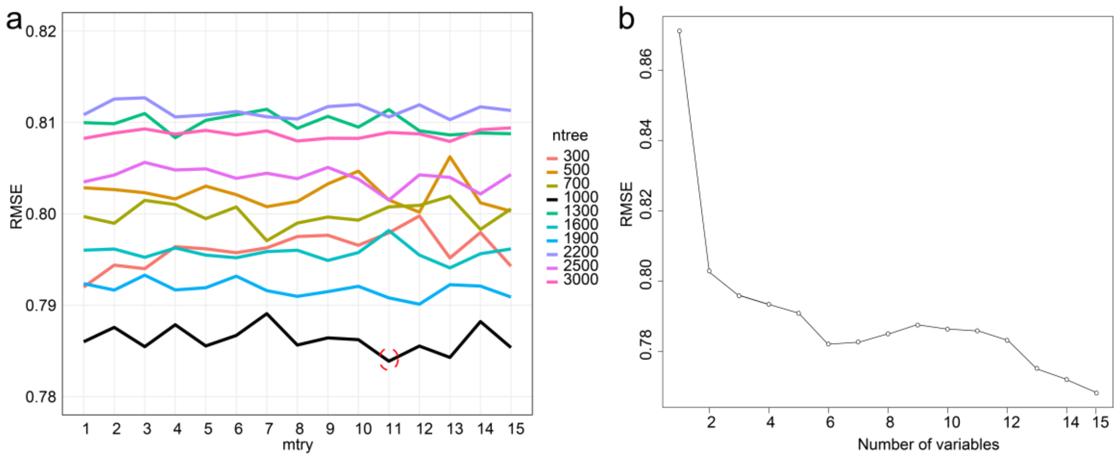

Supplementary Fig. 3 Hyperparameter tuning for random forest algorithm to predict the abundance of cobamide producers in soil environment based on grid search and 10-fold cross-validation. The best ntree and mtry are 1000 and 11, respectively (a). The following cross-validation shows the number of variables of 15 has the lowest cross-validated root mean square error (RMSE) value (b).

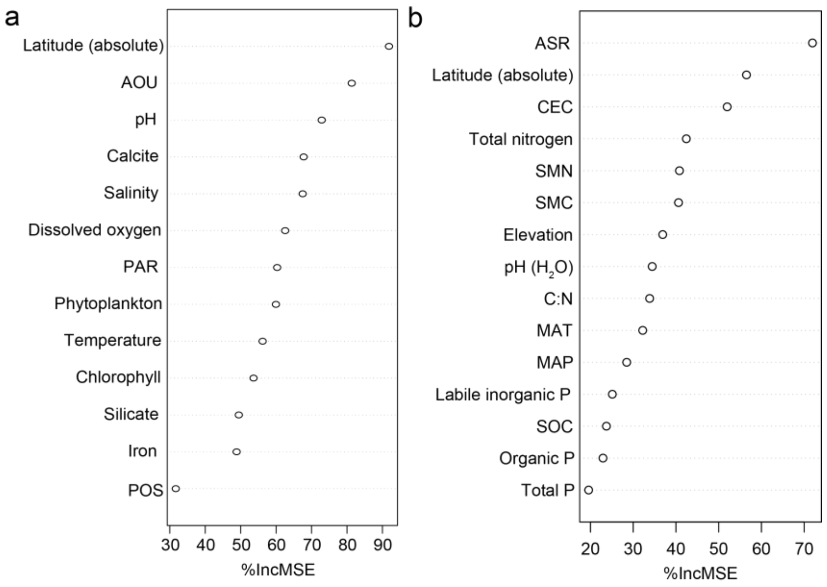

Supplementary Fig. 4 Optimal variables and relative importance for marine (a) and soil (b) random forest models. AOU: apparent oxygen utilization, PAR: photosynthesis available radiation, POS: percent oxygen saturation, ASR: annual solar radius, CEC: cation exchange capacity, SMN: soil microbial biomass nitrogen, SMC: soil microbial biomass carbon, MAT: mean annual temperature, MAP: mean annual precipitation, Labile inorganic P: labile inorganic phosphorus, SOC: soil organic carbon, Organic P: soil organic phosphorus, Total P: soil total phosphorus.

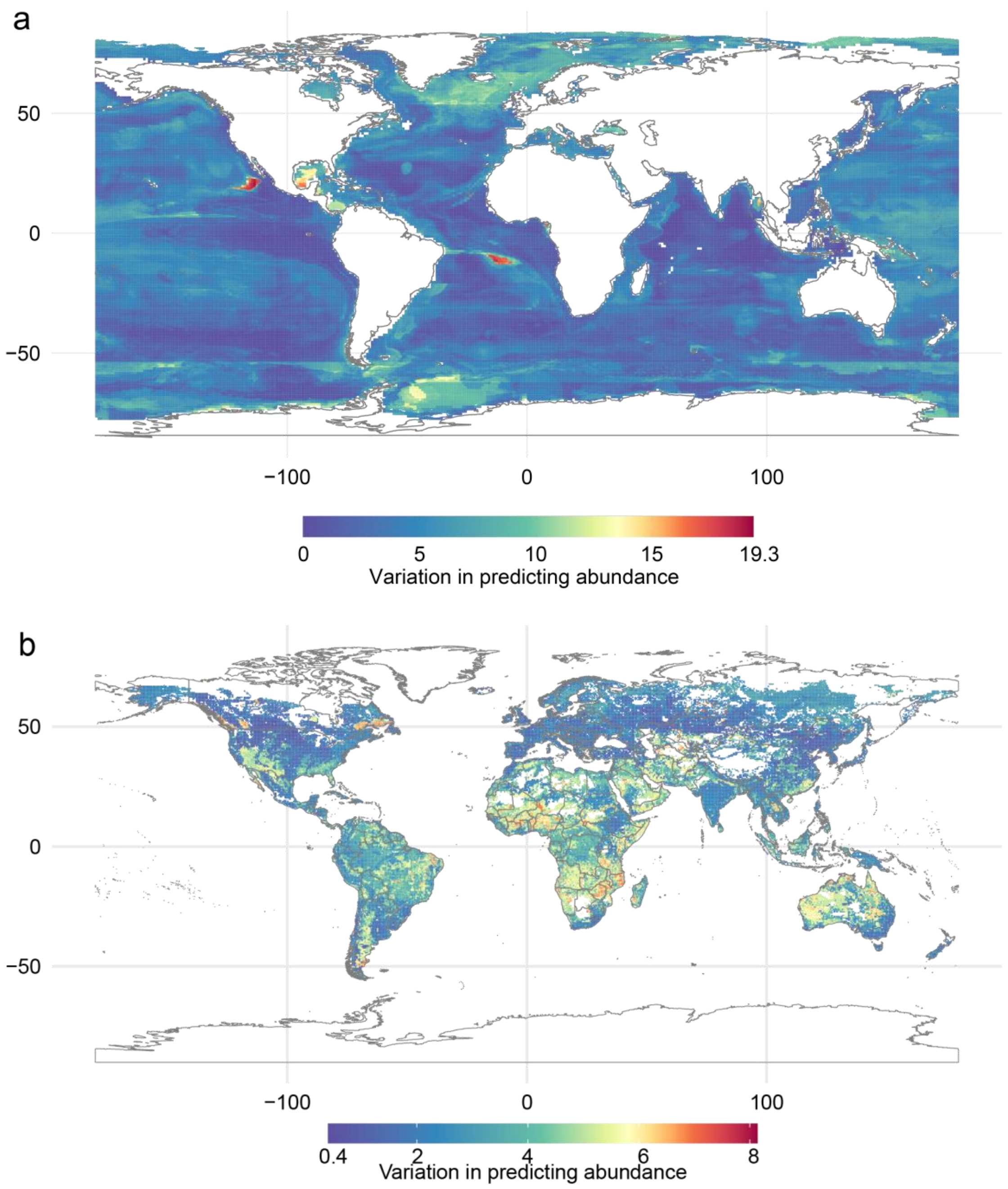

Supplementary Fig. 5 Uncertainty of predicted abundance of cobamide producers using coefficient of variation of random forest algorithm for marine (a) and soil (b) environments.

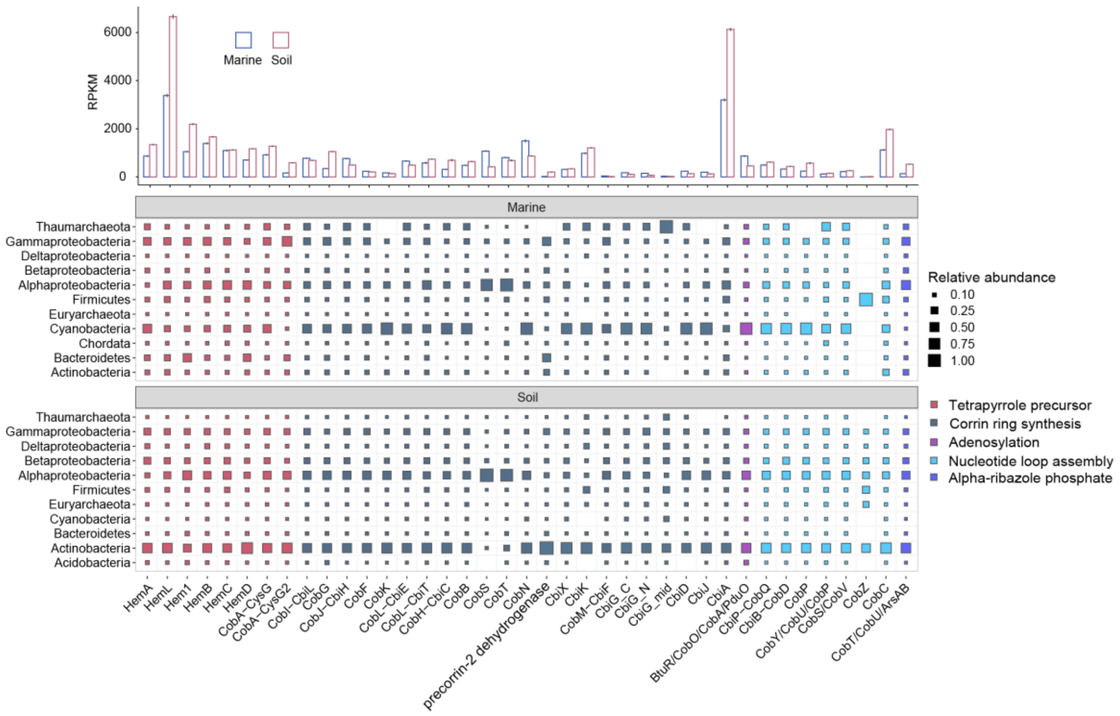

Supplementary Fig. 6 The biosynthesis gene abundance of top 8 prokaryotic phyla of samples from marine (upper) and soil (lower). Proteobacteria is divided into its classes. Genes are color-coded according to the five biosynthetic pathways. The bar plot at the top panel indicates the reads per kilobase of exon model per million mapped reads (RPKM) of genes; values are presented as mean ± standard error. “CobA-CysG” and “CobA-CysG2” refer to the same gene but correspond to different Hidden Markov Model (HMM) profiles of TIGR01469 and TIGR01470, respectively.

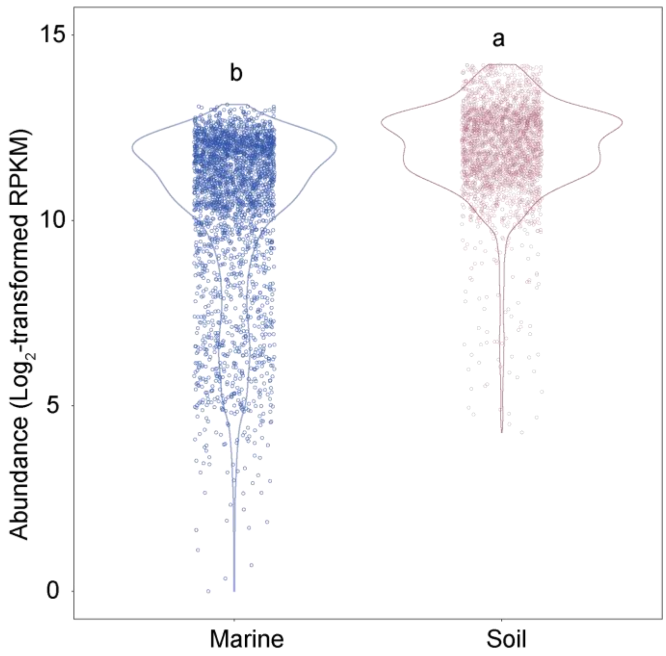

Supplementary Fig. 7 Abundance of cobamide producers in marine and soil environments. Different letters indicate significant difference (Wilcoxon test's $P < 0.001$). Potential biases were mitigated by excluding outliers once only. Samples in deep marine layers (> 100 m) and soil layers (> 30 cm) are not included.

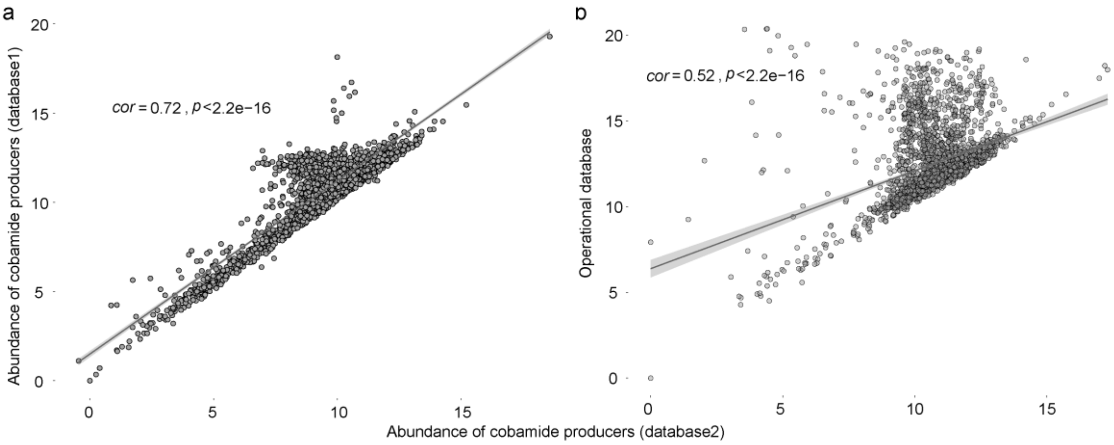

Supplementary Fig. 8 Spearman's correlation between the abundance of cobamide producers using two database in marine (a) and soil (b) environments. Database1 indicates using the MAGs with quality score higher than 50, database2 indicates using the MAGs with completeness higher than 90% and containment less than 5%.

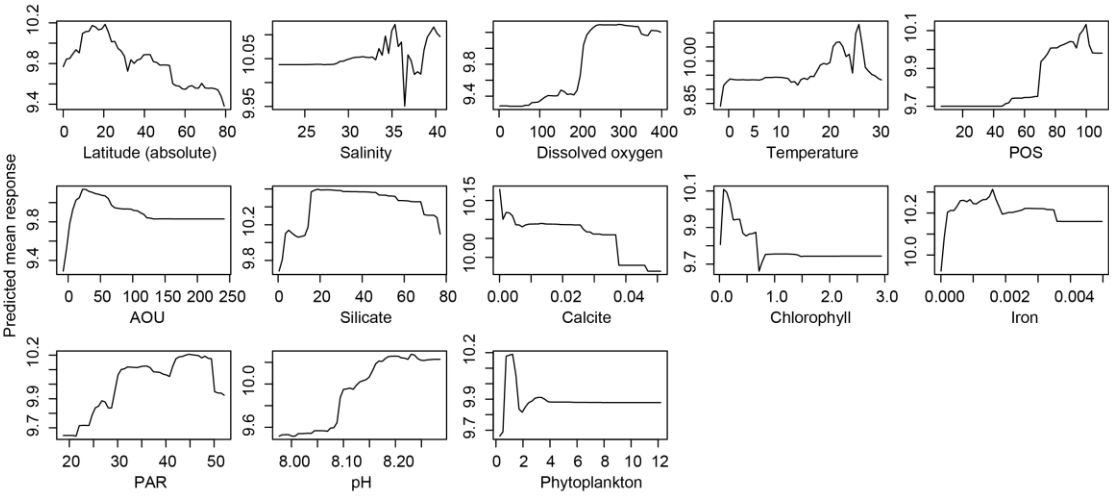

Supplementary Fig. 9 Partial dependence plot for random forest modeling based on abundance of cobamide producers (Log_2-transformed) of surface marine samples. POS: percent oxygen saturation, AOU: apparent oxygen utilization, PAR: photosynthesis available radiation.

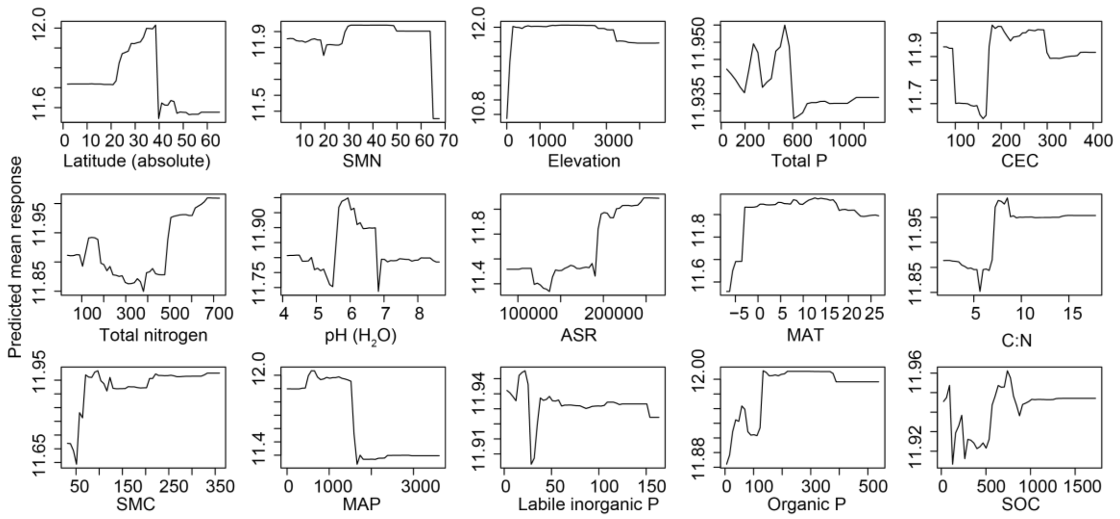

Supplementary Fig. 10 Partial dependence plot for random forest modeling based on abundance of cobamide producers (Log_2-transformed) of surface soil samples. SMN: soil microbial biomass nitrogen, Total P: soil total phosphorus, CEC: cation exchange capacity, ASR: annual solar radius, MAT: mean annual temperature, SMC: soil microbial biomass carbon, MAP: mean annual precipitation, Labile inorganic P: labile inorganic phosphorus, Organic P: soil organic phosphorus, SOC: soil organic carbon.

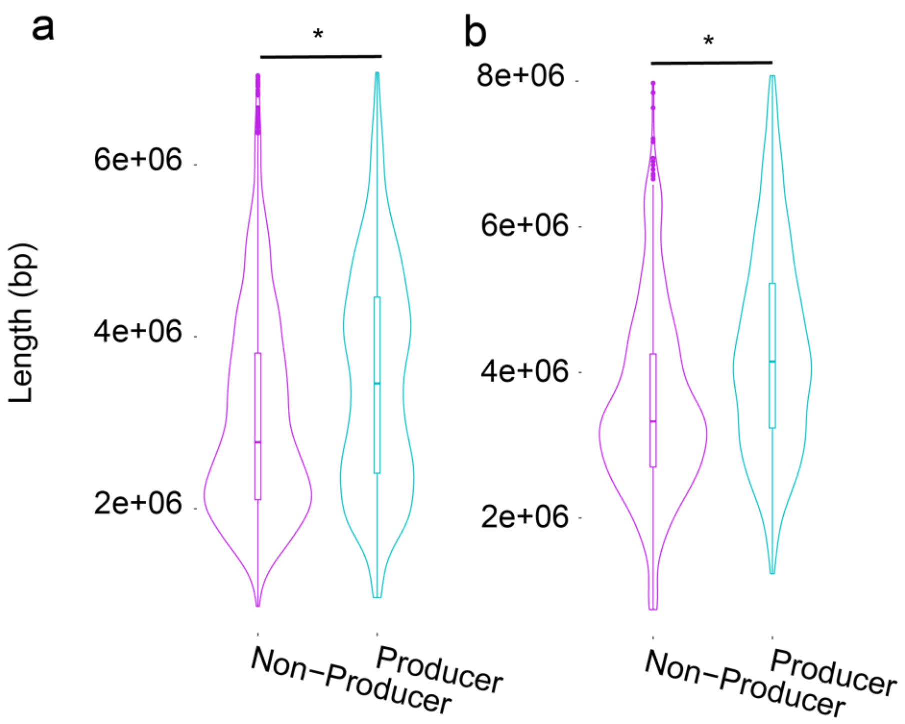

Supplementary Fig. 11 Size of Metagenome-Assembled Genome (MAG) identified as potential cobamide producer or non-producer of marine (a) and soil (b). Outliers are excluded once only. * indicates significant difference between these two groups (Wilcoxon test's $P < 0.01$).
